# Aspiration in Treatment of Hernia

**Published:** 1877-04

**Authors:** 


### Aspiration in the Treatment of Hernia.

Mr. P. L. O'Neill, Medical Officer, Athy Workhouse, writes to the *Medical Press and Circular:*

The following case is illustrative of the suecess which Dr. Dieulafoy claims for the aspirator as an aid to the taxis in the reduction of hernia, and likewise of the complete harmlessness of one or more punctures in the intestines, even of patients having a peculiar liability to serous inflammation :

On Sunday, the 19th inst., a patient, far advanced in Bright's disease of kidney, and who had, for some years, a small hernial protrusion, for which he usually wore a truss, was admitted to the Infirmary under the following circumstances, viz : Immediately before his admission, while lifting a weight in the absence of the truss, the hernia, which had hitherto confined itself to the region of the external abdominal ring, and was not at any time larger than a pigeon's egg, fell into the scrotum, and in a few moments became as large as a foot-ball, assuming the most exquisite tenderness. I saw him a few hours after the occurrence, but he could not permit me to touch the tumor. To allay the pain, I injected a quarter of a grain of acetate of morphia subcutaneously, and had warm fomentations and a stimulating enema administered.

One hour afterward I revisited patient, and found pain and tenderness abated, but manifested symptoms of strangulation, without any diminution in the size of the tumor. I applied the taxis for ten minutes without success ; then had patient placed in a warm bath, and repeated the taxis, with no better result. I next resolved on giving Dieulafoy's method a fair trial, and accordingly aspirated the hernia with a hot needle, with very satisfactory results. I then replaced it with a No. 2, which brought away some reddish fluid, fecal contents and flatus ; the needle, however, becoming clogged, I withdrew, cleaned and re-inserted it in another part of the tumor, with the most satisfactory results. Large quantities of flatus were extracted, the hernia reduced to less than half the size it had been a few moments before, and the merest effort at the taxis placed the bowel within the abdomen. From that time to this (Saturday, 25th Nov.) patient has had a stool daily, and not the smallest inconvenience in the abdomen.

The features in the case most worthy of remark are :—1st. That morphia, subcutaneously injected, was preferred to the administration of chloroform, which I would have considered dangerous, owing to the renal affection and cardiac weakness. 2d. That patient escaped peritonitis, notwithstanding the peculiar liability such persons have to inflammations. 3d. The facility with which the tumor was reduced after aspiration.—*Med. & Surg. Reporter.*

